# Immune Basis of Therapeutic Effects of *Solanum tuberosum* L. Polysaccharide on Chronic Peptic Ulcer Healing

**DOI:** 10.3390/ph18040502

**Published:** 2025-03-31

**Authors:** Evgenii Generalov, Denis Laryushkin, Kristina Kritskaya, Nina Kulchenko, Arkady Sinitsyn, Leonid Yakovenko, Liubov Generalova, Nikolay Belostotsky

**Affiliations:** 1 Faculty of Physics, Lomonosov Moscow State University, 119991 Moscow, Russia; yakovenko.lv@physics.msu.ru; 2 Federal Research Center “Pushchino Scientific Center for Biological Research of the Russian Academy of Sciences”, Institute of Cell Biophysics of the Russian Academy of Sciences, 142290 Pushchino, Russia; mr.ldp@yandex.ru (D.L.); kritskayak96@yandex.ru (K.K.); 3 Scientific and Educational Resource Center for Innovative Technologies of Immunophenotyping, Digital Spatial Profiling and Ultrastructural Analysis of the RUDN University, 117198 Moscow, Russia; kle-kni@mail.ru; 4 Department of Tumors of the Reproductive and Urinary Organs of Oncourology P.A. Hertsen Moscow Oncology Research Institute—Branch of the National Medical Research Radiological Centre, 125284 Moscow, Russia; 5 Faculty of Chemistry, Lomonosov Moscow State University, 119991 Moscow, Russia; apsinitsyn@gmail.com; 6 Federal Research Centre ‘Fundamental of Biotechnology’ of the Russian Academy of Sciences (FRC Biotechnology RAS), 119071 Moscow, Russia; 7 Faculty of Medicine, RUDN University, 117198 Moscow, Russia; generals1100@mail.ru; 8 A.S. Loginov Moscow Clinical Research and Practical Center of the Department of Health of Moscow, 111123 Moscow, Russia; belostotsky-pathophysiol2013@yandex.ru

**Keywords:** *Solanum tuberosum L.* polysaccharide, peptic ulcer, inflammation, Okabe model

## Abstract

**Background/Objectives**: Polysaccharides are complex molecules with a wide range of biological activities that can be used in various biomedical applications. In this work, the antiulcer effect and influence on the level of pro- and anti-inflammatory cytokines of *Solanum tuberosum L*. polysaccharide (STP) were studied. **Methods**: The antiulcer effect of STP was studied in the Okabe chronic peptic ulcer model by evaluating the influence of STP on the ulcer index in Wistar rats, comparing it to omeprazole and ranitidine. Dose-effect analysis was also carried out. The level of pro- and anti-inflammatory cytokines was studied using ELISA kits. **Results**: After treatment in the polysaccharide groups, ulcer healing is observed in 60–80% of cases, in the omeprazole group in 50%, and in the ranitidine group in 25%. STP intravenous injections lead to the formation of a more differentiated mucous membrane; no coarse scar tissue is formed, which is typical for control and comparison drugs. Glycan causes a significant acceleration of the healing of experimental peptic ulcers in rats. STP appears to modulate pro- and anti-inflammatory cytokines. On the fourth and tenth days, a significant decrease in the levels of pro-inflammatory cytokines IL-1b and IFN-γ was noted in the polysaccharide group compared to the control group, while the level of anti-inflammatory cytokine IL-4 significantly increased. **Conclusions**: Intravenous administration of STP leads to the restoration of functionality and effective tissue regeneration. The antiulcer activity of STP is based on the regulation of the pro- and anti-inflammatory balance.

## 1. Introduction

Peptic ulcer (PU) is a prevalent inflammatory condition characterized by a significant disruption of the gastric mucosa. Annually, millions are diagnosed with PU globally, with its prevalence estimated between 5 and 10% of the general population, depending on geographical features [1]. The condition is particularly concerning due to its notable recurrence rate, which can reach as high as 31%, coupled with adverse effects associated with conventional treatment modalities. This underscores the necessity for novel therapeutic strategies that include the development of pharmaceutical agents with a reduced negative side effect profile (for instance, fever, nausea, severe diarrhea, risk of developing precancerous conditions, jaundice, constipation, etc.) [2,3].

Polysaccharides are complex carbohydrates made up of long monosaccharide chains and play an important role in human nutrition and health [4,5,6]. One of their significant features is their anti-ulcer properties, which are due to several mechanisms of action. Anti-inflammatory properties of polysaccharides can be used to reduce inflammation in the gastrointestinal tract and stimulate healing processes by activating cell migration and proliferation, improving microcirculation [7,8].

Recent investigations have increasingly focused on the therapeutic potential of polysaccharides, which exhibit a broad array of biological activities [6]. Polysaccharides derived from fungi, algae, and higher plants are of great interest due to their immunomodulatory, anti-inflammatory, antitumor, radioprotective [9,10], wound healing [11], antiulcer [12], antiviral [13,14], and antiatherosclerotic properties. Notably, polysaccharide-based immunomodulators have the capacity to enhance innate immune responses against various pathogens [15,16,17,18]. The low toxicity and minimal side effects of plant-derived polysaccharides render them particularly advantageous compared to synthetic and microbial immunomodulators.

There are several common and well-known drugs for peptic ulcer treatment. H2 receptor antagonists, such as ranitidine and famotidine, were once primary antiulcer medications. These drugs work by blocking histamine receptors in the stomach, thereby reducing acid production, but their acid suppression is generally less potent and lasts less time than proton pump inhibitors (PPIs). Some individuals may find that symptoms such as heartburn and abdominal pain persist despite regular dosing. While usually well tolerated, common side effects may include headache, dizziness, and digestive upset [19]. Additionally, the effectiveness of H2 receptor antagonists can sometimes wane over time, requiring dose adjustments or shifts in treatment strategy. Ultimately, their moderate acid-reducing properties mean that complete symptom relief or ulcer healing is not always achieved, making them a less reliable option compared to more potent alternatives for some cases [20].

Another well-known and widely used drug is omeprazole. While omeprazole is a common antiulcer drug, its effectiveness is not always guaranteed for every patient. As a PPI, it reduces stomach acid, but some individuals may still experience persistent symptoms despite treatment. This can occur due to different factors, such as underlying conditions not solely related to acid production. Though generally well tolerated, side effects, such as headaches, nausea, and diarrhea, can appear [21]. Moreover, long-term omeprazole use has been linked to potential risks, such as bone fractures and nutrient deficiencies, raising concerns about its suitability for extended periods [22]. Ultimately, despite its widespread use, omeprazole’s effectiveness can be limited in certain cases, necessitating alternative or adjunctive therapies.

Potato tubers, specifically *Solanum tuberosum L*., while predominantly recognized as a nutritional staple, have a longstanding history of medicinal applications. Considering its low cost, availability, previously studied biological activity, usage in traditional medicines, and biological safety of the raw material, *Solanum tuberosum L*. seems to be one of the ideal candidates for obtaining biologically active substances. Nevertheless, research on the immunomodulatory effects of polysaccharides extracted from *Solanum tuberosum* L. (STP) remains limited [23,24]. In several European countries, potato extracts are used therapeutically for gastrointestinal pathologies due to their anti-inflammatory properties [25,26]. Prior studies have demonstrated that these polysaccharides possess significant antiulcer activity, thereby establishing the importance of the present study as a logical progression of earlier research efforts [27,28]. It is very important to understand the immunological basis of the regenerative–reparative effects of polysaccharides in peptic ulcer disease. The main objective of this work was to determine the effect of the polysaccharide on the ulcer and levels of pro- and anti-inflammatory cytokines during the regeneration of the ulcer defect.

## 2. Results

### 2.1. STP in Chronic Peptic Ulcer Model

The first objective of our study was to investigate the effect of different doses of STP on the ulcer index in Wistar rats in an experimental model of chronic peptic ulcer. Taking into account the cyclic nature of peptic ulcer disease with periods of exacerbation on days 1, 4, 7, and 10 after induction, as well as our previously obtained data on the antiulcer effect of the drug, we developed a scheme for administering STP corresponding to these critical points in the development of the ulcer process. The drug was administered in doses of 100, 500, and 1000 µg/200 g of animal body weight on those days after ulcer induction. This approach allowed us not only to evaluate the dose-dependent effect of STP but also to study its effect at various stages of the ulcer process. Administration of the drug on days corresponding to the peaks of exacerbation allows us to evaluate its ability to prevent or reduce pathological changes in the mucous membrane, which is important for the potential clinical use of STP in the future.

**Figure 1:**
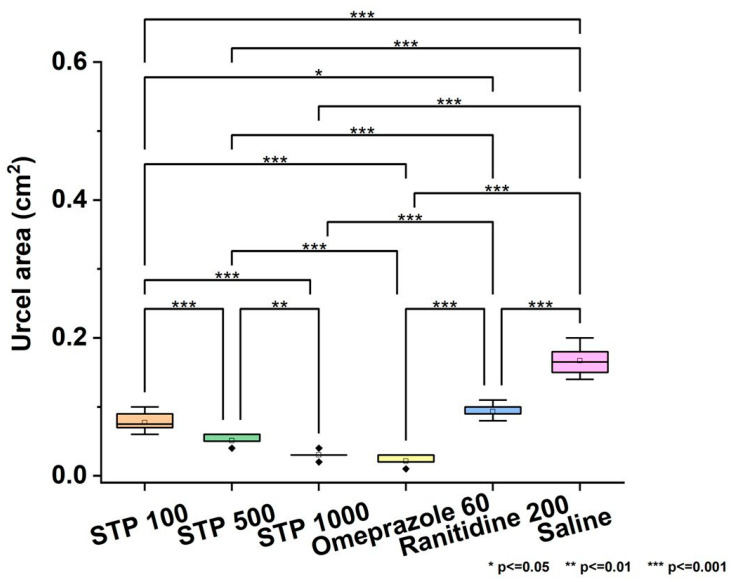
Effect of different doses of STP (100, 500, and 1000 µg/200 g, omeprazole 60 µg/200 g, ranitidine 200 µg/200 g, and physiological solution (saline) on the ulcer index in experimental animals. Data are presented as box plots, where the central line indicates the median and the upper and lower boundaries indicate the 25th and 75th percentiles, respectively. Statistical analysis was performed using the nonparametric Kruskal–Wallis test, followed by Bonferroni correction for multiple comparisons. A significant decrease in the ulcer index was observed in the groups receiving polysaccharides, especially the maximum doses and standard antiulcer drugs (omeprazole and ranitidine) compared to the control group (saline). Statistically significant differences are indicated by asterisks (*** *p* < 0.001).

As can be seen from Figure 1, the polysaccharide STP at doses of 100, 500, and 1000 µg/200 g significantly reduced the ulcer index compared to the control group (saline), which indicates its pronounced antiulcer effect. Moreover, the effectiveness of STP at a dose of 100 µg/200 g was comparable to the effect of standard antiulcer drugs—omeprazole and ranitidine—and at higher doses, it surpassed them. It is interesting to note that a dose-dependent effect was observed; with an increase in the STP dose from 100 to 1000 µg/200 g, the ulcer index consistently decreased. The ulcer index in Group 1 has the lowest value of 0.02 cm^2^, in Group 2, 0.03 cm^2^, and in Group 3, 0.06 cm^2^, which is several times less than in the control group.

### 2.2. STP «Dose–Effect» in Peptic Ulcer Model

Next, we calculated a dose–response curve to evaluate the efficacy of the drug at different doses.

**Figure 2:**
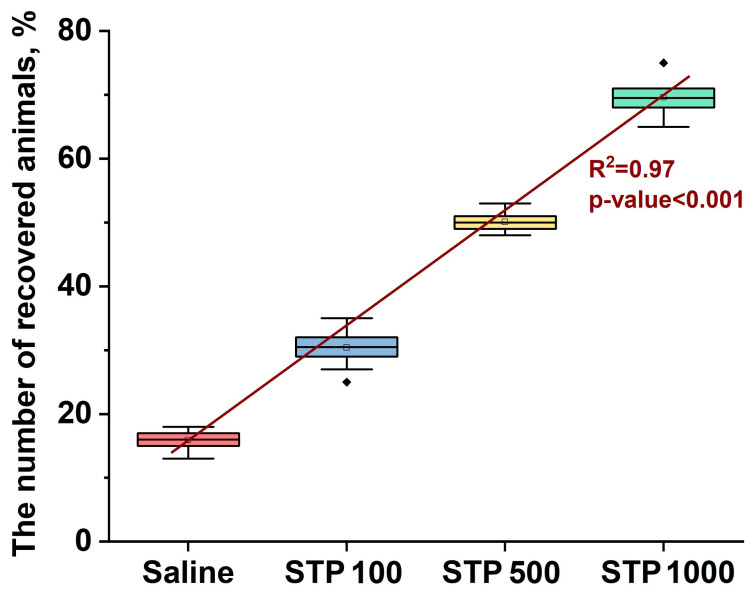
Dose–response analysis for STP. The graph shows the percentage of animals recovered as a function of the dose of the drug. The red line represents the linear trend. The rectangles show the distribution of data for each dose, where the horizontal line inside the rectangle is the median, the rectangle boundaries are the first and third quartiles, and the vertical lines are the minimum and maximum values. R^2^ = 0.97 indicates a strong correlation between dose and effect; *p* < 0.001 confirms the statistical significance of the results.

Figure 2 shows that there is a clear positive relationship between the dose of STP and the percentage of recovered animals. With an increase in the STP doses from absence (injection of saline solution) to 1000 µg/200 g, a significant and consistent increase in the proportion of animals with complete epithelialization of the ulcer defect was observed. The linear trend describes this relationship well, which is confirmed by the high value of the determination coefficient R^2^ = 0.97. The statistical significance of this relationship is confirmed by the extremely low *p* value (*p* < 0.001), indicating high reliability of the results obtained.

### 2.3. Effect of STP on the Balance of Pro- and Anti-Inflammatory Cytokines

**Figure 3:**
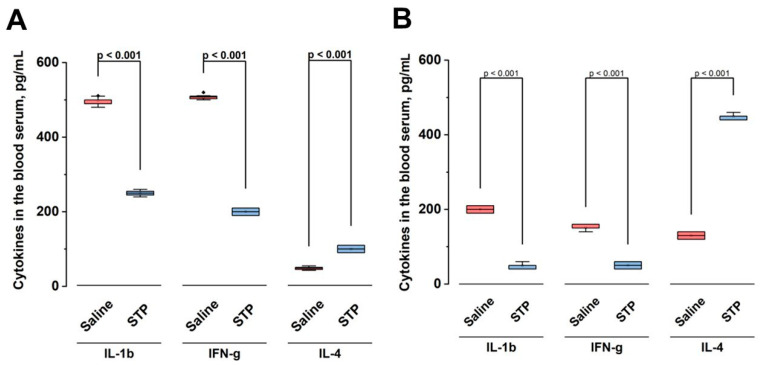
The effect of 500 µg/200 g STP on serum cytokine levels in experimental animals. (**A**) Cytokine levels on day 4 and (**B**) cytokine levels on day 10 of the experiment. Concentrations of IL-1b, IFN-γ, and IL-4 (pg/mL) in the main groups receiving saline and STP are shown. Boxplots show the distribution of data: the horizontal line inside is the median, the rectangle boundaries are the first and third quartiles, and the vertical lines are the minimum and maximum values. The analysis was performed using the nonparametric Kruskal–Wallis test. Statistical significance of differences between groups is indicated as *p* < 0.001 for all comparisons.

Figure 3 illustrates the dynamics of changes in the levels of key cytokines in the blood serum of experimental animals after the administration of STP at a dose of 500 µg/200 g compared to the control group receiving saline. Figure 3A shows the levels of cytokines on the fourth day of the experiment and Figure 3B on the 10th day.

A pronounced and statistically significant (*p* < 0.001) change in the concentrations of both pro- and anti-inflammatory cytokines is observed upon administration of STP at both time points. On the fourth day (Figure 3A), a significant decrease in the levels of pro-inflammatory cytokines IL-1b and IFN-γ was noted in the STP group compared to the control group, while the level of anti-inflammatory cytokine IL-4 significantly increased.

A similar trend was maintained on the 10th day of the experiment (Figure 3B), indicating a stable anti-inflammatory effect of STP. These results indicate the ability of STP to modulate the immune response, reducing the severity of the inflammatory reaction and stimulating anti-inflammatory mechanisms over a long period of time.

**Figure 4:**
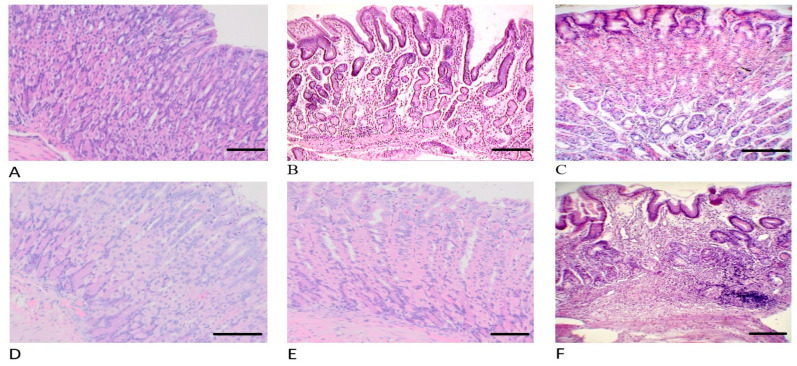
Histology of the rats’ stomachs on the 10th day after ulceration by the Okabe method. Hematoxylin and Eosin staining, ×200 magnification. (**A**) Group 1 with introduction of STP 1000 µg/mL/200 g. (**B**) Group 2 with the introduction of STP 500 µg/mL/200 g. (**C**) Group 3 with the introduction of STP 100 µg/mL/200 g. (**D**) Group 4 with the introduction of omeprazole 60 µg/200 g. (**E**) Group 5 with the introduction of ranitidine 200 µg/200 g. (**F**) Group 6 with the introduction of physiological solution (saline).

**Figure 5:**
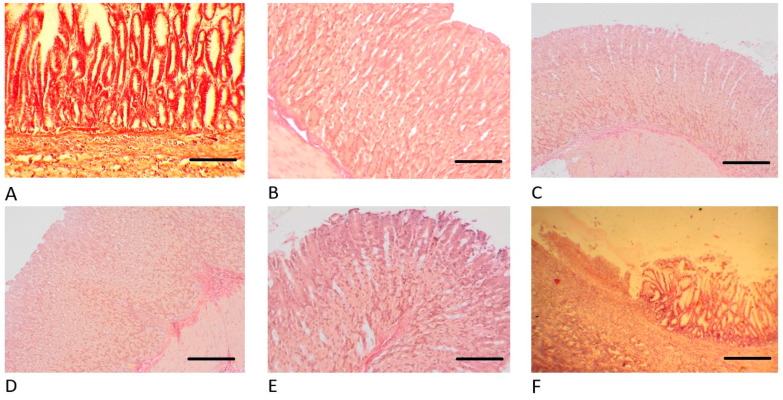
Histology of the rats’ stomachs on the 10th day after ulceration by the Okabe method. Van Gieson staining, ×200 magnification. (**A**) Group 1 with introduction of STP 1000 µg/mL/200 g. (**B**) Group 2 with the introduction of STP 500 µg/mL/200 g. (**C**) Group 3 with the introduction of STP 100 µg/mL/200 g. (**D**) Group 4 with the introduction of omeprazole 60 µg/200 g. (**E**) Group 5 with the introduction of ranitidine 200 µg/200 g. (**F**) Group 6 with the introduction of physiological solution (saline).

**Figure 6:**
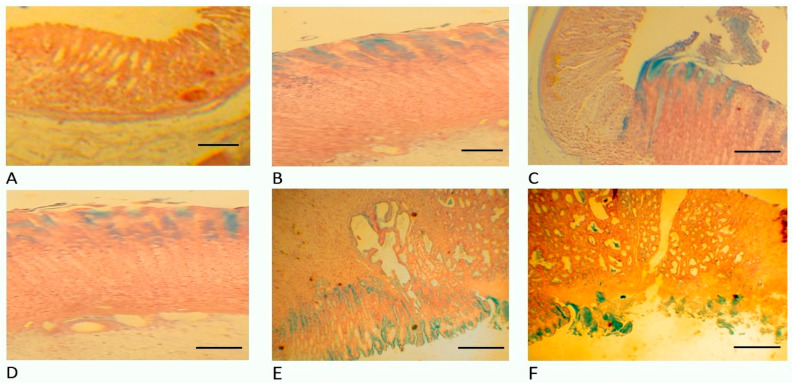
Histology of the rats’ stomachs on the 10th day after ulceration by the Okabe method. Alcian blue staining, ×200 magnification. (**A**) Group 1 with introduction of STP 1000 µg/mL/200 g. (**B**) Group 2 with the introduction of STP 500 µg/mL/200 g. (**C**) Group 3 with the introduction of STP 100 µg/mL/200 g. (**D**) Group 4 with the introduction of omeprazole 60 µg/200 g. (**E**) Group 5 with the introduction of ranitidine 200 µg/200 g. (**F**) Group 6 with the introduction of physiological solution (saline).

When examining the surface of the gastric mucosa ad oculus, we did not find areas of mucosal necrosis in any animal in the STP experimental group. Histological analysis (Figure 4, Figure 5 and Figure 6) of gastric wall specimens confirmed that there were no ulceration areas of gastric mucosa in the groups of animals receiving STP (especially in groups 1 and 2). In those places where it was possible to visualize the bottom of the ulcer defect, there were no necrotic masses. The forming granulations contain a greater number of newly formed vessels and more mature fibroblasts than in the groups of saline, omeprazole, and ranitidine. In addition, in the control group, with the introduction of saline, there were many areas of damage to the mucous membrane, with signs of fibrinoid necrosis.

In the experimental groups, within the lamina propria of the mucosa, fibroblasts were oriented in a perpendicular direction; in the submucosa, they were located parallel to the surface of the mucosa. A positive reaction with alcian blue indicates the presence of mucins and acidic glycosaminoglycans at the edges of the ulcer, which clearly demonstrates the healing phenomena manifested in the form of “crawling” of flattened epithelium saturated with RNA onto the surface of the ulcer bottom and the formation of a pyloric-type mucous membrane, despite the fact that the ulcer is located in the fundus. In all animals that received STP injections, the gastric pits were deep and lined with epithelium with signs of basophilia. Distally, the epithelium took the form of a highly prismatic one containing many acidic and neutral glucosaminoglycans. That is, in the rats of the experimental groups, regeneration of the inner lining of the stomach was observed according to the type of formation of the fundic mucosa; the main and parietal cells appear.

In the submucosa of the animals of the main groups, numerous delicate, thin collagen fibers and fibroblasts were found. All these signs indicate active regeneration of the damaged stomach lining and faster, more reliable healing.

A similar morphological picture in the gastric biopsies was observed in the group with the introduction of omeprazole; a macroscopic and microscopic examination of the mucosa also did not reveal areas of necrosis of the gastric mucosa. In all cases, signs of ulcer healing were found, but less actively, which is confirmed by dystrophy of epithelial cells, cystic transformation of glands, moderate leukocyte infiltration, the absence of signs of formation of the main glands, fibrosis of the proper plate of the mucous and submucosa up to the formation of local scars.

In the control group (with the introduction of a physiological solution), the gastric mucosa had many ulcers. The ulcers had an extensive bottom covered with necrotic masses, under which granulation tissue, collagen fibers, and vascular loops were poorly expressed. Many cysts and dilated glands were observed at the edges of the ulcer, while there were no signs of the formation of the main glands against the background of the formation of coarse scar tissue.

A similar histological picture in the group with the injection of ranitidine has been observed.

In none of the experimental Groups 1–5 were negative side effects observed.

All these data indicate that STP causes a significant acceleration of the healing of experimental “chronic” “acetate” peptic ulcers in rats in comparison with a proton pump inhibitor (omeprazole), a blocker of histamine H2 receptors of parietal cells of the gastric mucosa (ranitidine), and physiological solution.

Ulcer healing implies complete epithelialization of its surface, formation of highly differentiated, full-fledged pyloric mucosa, and restoration of submucosa and muscular membranes to the depth of the entire ulcer defect. Our study showed that after 9 days of treatment in STP groups, ulcer healing is observed in 60–80% of cases, in the omeprazole group in 50%, and in the ranitidine group in 25%. We associate such a significant positive difference in the regeneration of the “chronic” “acetate” ulcer against the background of STP treatment with satisfactory epithelialization of the mucosa in the absence of fibrosis in the mucosa lamina propria and submucosa, which facilitates the formation of new vessels at the site of the ulcer defect and improves tissue trophism. There were no visible negative side effects of STP treatment.

## 3. Discussion

This study demonstrated the therapeutic effect of STP injections on the treatment of peptic ulcers. The results demonstrate that STP effectively reduces the ulcer index and outperforms standard drugs such as omeprazole and ranitidine, especially when used in higher doses of 500 and 1000 µg/mL. These data are consistent with previous studies describing the anti-inflammatory and regenerative properties of polysaccharides that have a protective effect on the gastric mucosa [29,30]. The observed dose-dependent effect of STP indicates the possibility of its use in various clinical scenarios, since even the lowest dose of STP (100 µg/200 g) demonstrated an efficacy comparable to traditional drugs, while higher doses (500 and 1000 µg/200 g) exceeded them. These results highlight the flexibility in choosing STP dosages, which may be useful for adapting therapy to the severity of the disease and the individual needs of patients. Dose–response analysis (R^2^ = 0.97, *p* < 0.001) confirmed a positive correlation between the STP dosage and the percentage of recovered animals, indicating the predictability of the therapeutic effect when changing the dosage. This is an important factor in the development of individualized therapeutic protocols, where dose changes can be applied to achieve an optimal clinical outcome.

The effect of STP on the balance of pro- and anti-inflammatory cytokines was significant. A decrease in IL-1β and IFN-γ levels combined with an increase in IL-4 levels indicates its potent anti-inflammatory effect, which probably plays a key role in preventing peptic ulcer relapses. This effect was maintained throughout the observation period, confirming the long-term effect of STP on the regulation of the immune response, which may be useful in the prevention of chronic inflammatory diseases of the stomach.

Histological analysis showed that STP treatment resulted in a significant improvement in tissue regeneration compared to the control groups. In particular, an increase in the number of newly formed vessels and mature fibroblasts was observed, indicating high-quality regeneration. In addition, the absence of necrotic masses and cystic formations during STP treatment indicates its advantage over omeprazole and ranitidine, which may reduce the risk of relapses and cancer development associated with pathological tissue changes [31].

We suggest that the mechanism of action of STP involves several factors. In addition to the direct anti-inflammatory effect through modulation of the cytokine profile, STP is likely to stimulate tissue regeneration processes, as evidenced by active vascular formation and fibroblast differentiation. Additionally, a positive reaction to Alcian blue staining indicates an increase in the production of mucins and acidic glycosaminoglycans, which strengthens the protective barrier of the gastric mucosa and promotes its restoration. These data are consistent with modern concepts of the role of the extracellular matrix in tissue healing processes [32]. The formation of pyloric-type mucosa in the fundus of the stomach was also observed with STP, which may indicate its ability to influence cellular differentiation and tissue architecture. These results are of interest for further studies since the ability to modulate tissue structures may find application in regenerative medicine.

Despite the positive results, the study has several limitations. First, the relatively small sample size and short follow-up period limit the extrapolation of the obtained data to the long-term clinical perspective. Second, the relatively small number of cytokines measured. For a better understanding of immunological processes, other cytokines have to be studied further at different time points.

The mechanism of action of STP, although demonstrating multifactoriality, requires additional study at the molecular level. Future studies, including gene expression analysis and proteomic studies, may elucidate the molecular mechanisms of action of STP, which will allow for a more accurate definition of its therapeutic targets. It should also be noted that, despite the focus of our study on gastric ulcer disease, the therapeutic potential of STP may extend to other gastrointestinal diseases, including inflammatory bowel disease and gastroesophageal reflux disease. Studying the efficacy of STP in these conditions could expand its therapeutic spectrum.

## 4. Materials and Methods

### 4.1. STP Activity in Okabe Model

To study the specific activity of STP (polysaccharide complex with molecular weight around 70 kDa, mainly consisting of arabinose and galactose, and other monosaccharides, such as glucose), which was extracted (extracted and purified from *Solanum tuberosum* L. using cross-flow, chromatography and membrane methods, obtained from LLC SPC Gemma-B) as described in the previous work [33], in the Okabe model [34] of the “chronic” “acetate” peptic ulcer model, we used Wistar female rats (from the branch of the M.M. Shemyakin and Yu.A. Ovchinnikov Institute of Bioorganic Chemistry of the Russian Academy of Sciences), weighing 190–210 g, with 10 animals in each group. STP was administered to rats on the first, fourth and seventh days into the tail vein at doses of 1000 µg (Group 1), 500 µg (Group 2) and 100 µg (Group 3)/200 g. Omeprazole 60 μg/200 g (Group 4), ranitidine 200 μg/200 g (Group 5) and saline (Group 6—control) were chosen as the controls. The comparison drugs were administered to the model animals daily in accordance with the instructions for their use. Saline, like STP, was administered on days 1, 4, and 7 in the same volume.

Under ether anesthesia, the abdominal cavity of the animals was accessed via a midline incision along the linea alba. The stomach was then gently exteriorized. A specialized ring (internal diameter = 0.5 cm) was carefully positioned and applied to the serosal surface of the anterior gastric wall, specifically within the corpus region. Precisely 0.03 mL of 100% acetic acid was then applied within the confines of the ring for a duration of 30 s. Following this exposure period, the area was meticulously blotted dry using sterile filter paper to remove any residual acid. During ring placement, care was taken to avoid major blood vessels. Finally, the abdominal wall was closed using a layered suture technique. Eighteen hours prior to the surgical intervention, the model animals were subjected to a fasting period with free access to water. Post-surgery, the animals were returned to a standard dietary regimen. The operated animals with a “chronic” ulcer were observed after the operation for 9 days, after which they were euthanized humanely using carbon dioxide on the 10th day.

In our study, we initially conducted a macroscopic evaluation of the mucosal surface using a magnifying lens. Subsequently, we quantified the ulcerative index employing the ImageJ 1.53 software package (Bethesda, MD, USA). The results were expressed as the total area of ulcerative lesions (cm^2^).

Upon conducting a macroscopic analysis of the gastric mucosa, a subsequent microscopic examination was undertaken. Fragments from the anterior wall of the gastric body were preserved in a 10% formalin solution and buffered in accordance with Lilly’s protocol (pH 7.2–7.4). The specimens underwent standard histological processing, followed by preparation of histological sections stained using hematoxylin and eosin, as well as van Gieson and Alcian blue techniques, all examined under light microscopy.

### 4.2. Effect of STP on Cytokine Level

To study the effect of STP on cytokine production in animals with a “chronic” ulcer, blood was taken from the tail vein on days 4 and 10 and analyzed according to the standard protocol of the manufacturers of Rt IL-1β, Rt IL-4 and Rt IFNγ ELISA kit (LS-F5627, LS Bio a Vector Laboratories, Inc., Newark, NJ, USA; ER0041, Wuhan Fine Biotech Co., Ltd, Wuhan, China; ab239425, Abcam Limited, Cambridge, UK).

### 4.3. Statistical Analysis

The effect of different doses of STP (100, 500, and 1000 µg/200 g), standard antiulcer drugs (omeprazole, ranitidine) and physiological solution (saline) on the ulcer area in experimental animals was analyzed. Data are presented as box plots, where the central line indicates the median and the upper and lower boundaries represent the 25th and 75th percentiles. Statistical analysis was performed using the nonparametric Kruskal–Wallis test followed by the Tukey test for multiple comparisons (significance level: *p* < 0.05). A significant decrease in ulcer area was observed in the groups receiving STP (especially at the maximum doses) and standard antiulcer drugs compared to the control group (saline). Statistically significant differences are indicated by asterisks (*** *p* < 0.001).

Statistical analysis was carried out using OriginLab 2024b. First, the data were checked for normality using the Shapiro–Wilk normality test. Then, groups were compared using the Kruskal–Wallis test followed by the Tukey test for multiple comparisons. To analyze the dose–effect relationship of STP, a graphical method was used to display the percentage of recovered animals depending on the drug dose. The data are presented as a box plot. To assess the strength of the relationship between the drug dose and the observed effect, the determination coefficient (R^2^) was calculated. An R^2^ value of 0.97 indicates a strong positive correlation between the dose and the effect, and the statistical significance of the results is confirmed by a value of *p* < 0.001.

All experimental protocols adhered strictly to the ethical principles outlined in the Helsinki Declaration of 1964 and its subsequent amendments. The animal studies were conducted in compliance with the Federation of European Laboratory Animal Science Associations (FELASA) guidelines for the ethical treatment and use of laboratory animals [35] and with the European Convention for the Protection of Vertebrate Animals (Strasbourg, 18 March 1986). These studies were approved by the Ethics Committee of the Institute of Cell Biophysics of the Russian Academy of Sciences, with permissions granted under Approval No. 4 on 14 March 2022 and Approval No. 3 on 12 March 2023. The animals were housed in individually ventilated cages, conforming to Directive 2010/63/EU of the European Parliament and Council, from 22 September 2010, which governs the protection of animals utilized for scientific purposes [36].

## 5. Conclusions

When STP is administered intravenously to laboratory animals, ulcerative defects heal due to the formation of a more differentiated mucous membrane. Microscopically, full-fledged glandular epithelium is formed, which means the restoration of functionality and effective tissue regeneration. A positive aspect of using STP in the treatment of “chronic” “acetate” ulcers is that, against the background of mucosal epithelialization, no coarse scar tissue is formed, which is typical for the control and comparison drugs: a proton pump inhibitor (omeprazole) and a histamine H2 receptor blocker (ranitidine).

It is also important that STP affects the levels of IL-1, IL-4, and IFN-γ cytokines. Modulation of pro- and anti-inflammatory cytokines, a significant decrease in pro-inflammatory cytokines and a decrease in their cytotoxic effect, with a simultaneous increase in anti-inflammatory cytokines, was observed. We believe it is important that this property of STP accelerates regenerative and reparative processes. In addition, tissue restoration is achieved by increasing the level of cell differentiation, improving the spatial organization of collagen fibers in the gastric submucosa, and enhancing the trophism of the damaged area as a result of the formation of additional blood vessels.

## Data Availability

The data presented in this study are available on request from the corresponding author.

## Abbreviations

The following abbreviations are used in this manuscript:

STP	*Solanum tuberosum* L. polysaccharide
PU	Peptic ulcer
IL	Interleukin
IFN	Interferon
PPI	Proton pump inhibitor
